# Methods for Evaluating Cell-Specific, Cell-Internalizing RNA Aptamers

**DOI:** 10.3390/ph6030295

**Published:** 2013-03-14

**Authors:** Luiza I. Hernandez, Katie S. Flenker, Frank J. Hernandez, Aloysius J. Klingelhutz, James O. McNamara II, Paloma H. Giangrande

**Affiliations:** 1 Department of Internal Medicine, University of Iowa, Iowa City, IA 52242, USA; E-Mails: luiza-hernandez@uiowa.edu (L.I.H); katie-stockdale@uiowa.edu (K.S.F); frank hernandez@uiowa.edu (F.J.H); james-mcnamara@uiowa.edu (J.O.M); 2 Department of Microbiology, University of Iowa, Iowa City, IA 52242, USA; E-Mail: al-klingelhutz@uiowa.edu; 3 Department of Radiation Oncology, University of Iowa, Iowa City, IA 52242, USA; 4 Molecular & Cellular Biology Program, University of Iowa, Iowa City, IA 52242, USA

**Keywords:** RNA aptamers, targeted delivery, siRNA delivery, cell-SELEX, cell-internalizing aptamers

## Abstract

Recent clinical trials of small interfering RNAs (siRNAs) highlight the need for robust delivery technologies that will facilitate the successful application of these therapeutics to humans. Arguably, cell targeting by conjugation to cell-specific ligands provides a viable solution to this problem. Synthetic RNA ligands (aptamers) represent an emerging class of pharmaceuticals with great potential for targeted therapeutic applications. For targeted delivery of siRNAs with aptamers, the aptamer-siRNA conjugate must be taken up by cells and reach the cytoplasm. To this end, we have developed cell-based selection approaches to isolate aptamers that internalize upon binding to their cognate receptor on the cell surface. Here we describe methods to monitor for cellular uptake of aptamers. These include: (1) antibody amplification microscopy, (2) microplate-based fluorescence assay, (3) a quantitative and ultrasensitive internalization method (*“QUSIM”*) and (4) a way to monitor for cytoplasmic delivery using the ribosome inactivating protein-based (RNA-RIP) assay. Collectively, these methods provide a toolset that can expedite the development of aptamer ligands to target and deliver therapeutic siRNAs *in vivo*.

## 1. Introduction

Since the discovery of RNA interference (RNAi), substantial efforts have been directed towards harnessing the sequence-specific silencing potential of small interfering (si)RNAs for therapeutic applications. Despite important advances in chemical modification strategies to enhance the stability of siRNAs in serum and reduce their off-target effects, delivery remains the most significant hurdle to the therapeutic application of siRNAs [1,2,3]. Effective use of RNAi *in vivo* depends on the delivery of exogenous siRNAs into the cytoplasm of target cells in quantities sufficient to silence gene expression and elicit the intended therapeutic effect. To this end, extensive research has focused on the development of targeted delivery systems capable of directing the siRNAs to the target cells and facilitating endosomal escape. Several of the existing delivery approaches make use of cell-specific targeting ligands or functional groups covalently conjugated to siRNAs to direct their delivery to a specific cell-type or tissue [2].

One such approach, based on synthetic RNA ligands (aptamers), has been employed by us and others, to facilitate delivery of siRNAs to the cytoplasm of target cells both *in vitro* and *in vivo* [4,5,6,7,8,9,10,11,12]. For this approach, aptamers that bind cell-specific receptors are conjugated with partially (one strand only) or fully (both strands) chemically-modified siRNAs in chimeric molecules. The aptamer directs the chimeric RNA (aptamer-siRNA conjugate) to the cells that express the aptamer-targeted receptor on their surface. The chimeric RNA is then internalized, released into the cytoplasm (by a mechanism that remains to be fully understood) and the siRNA is processed by the RNAi machinery, resulting in mRNA knockdown of the siRNA target gene selectively in the targeted cell population. We have pioneered this strategy for systemic administration of therapeutic anti-cancer siRNAs to mice bearing human-derived prostate tumors [4,5]. Since its conception, this strategy has been validated *in vivo* with systemic administration in xenograft mouse models of prostate cancer [5,10,11] and HIV-infected cells [9,12,13]. While the potential of this approach as a platform technology with broad applicability is substantial, its widespread adoption is contingent on the availability of aptamers to cell-surface receptors capable of entering and delivering their siRNA cargo to the cytoplasm of target cells.

**Figure 1:**
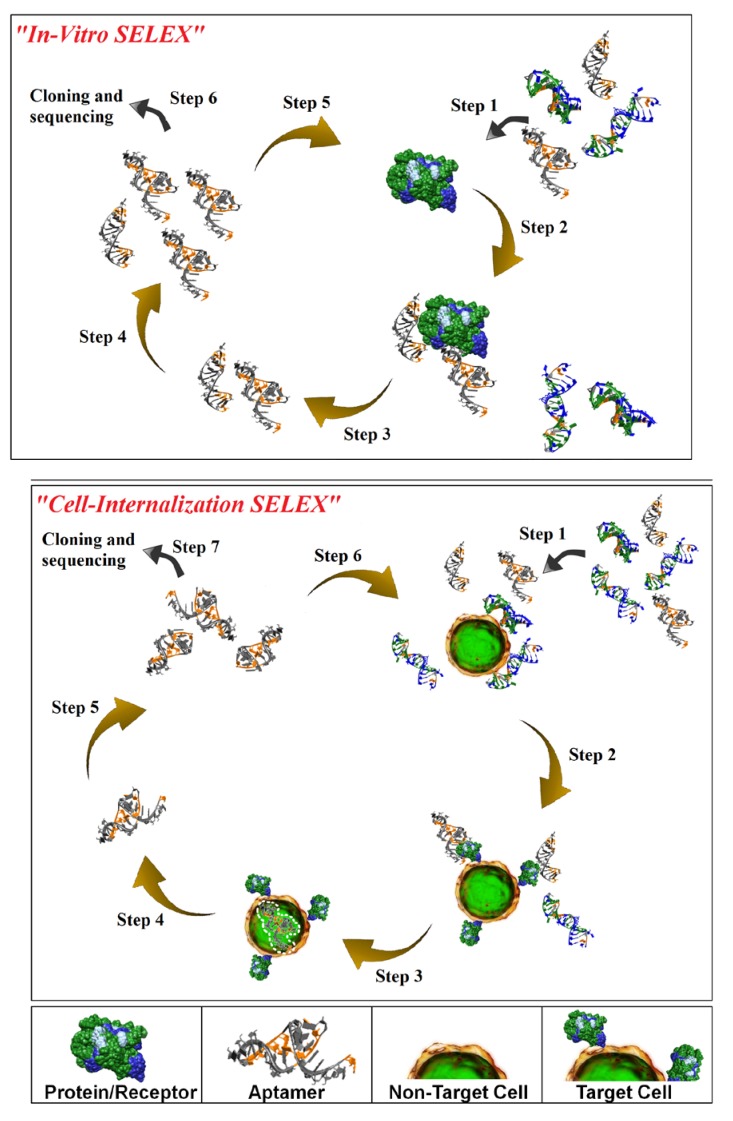
Schematic of *in vitro-*Systematic Evolution of Ligands by EXponential enrichment (SELEX; *upper panel*) and Cell-Internalization SELEX methodologies (*lower panel*). For *in vitro-*SELEX, a library of RNA molecules is incubated with the target (e.g. recombinant protein) (Step 1) and binders are separated from non-binders (Step 2). Recovered binders (Step 3) are subsequently amplified (Step 4) to generate a pool of molecules that displays high affinity and specificity for the target. For cell-internalization SELEX, a library of RNA molecules is incubated with non-target cells (counter selection) (Step 1) and those RNAs that do not bind (supernatant) are then transferred to the target cells (Step 2). The cell incubations are performed at 37 °C to facilitate cell internalization (Step 3). A stringent wash step (high salt wash, trypsin wash) is then performed to remove unbound and/or surface bound RNA. The internalized RNA is recovered using TRIzol extraction (Step 4) and amplified by PCR (Step 5). The process is repeated several more times to enrich for RNA sequences with cell-specific, internalizing properties (Step 6). The enriched pool of RNA is then subjected to cloning and sequencing (Step 7).

Isolation of aptamers with affinity and specificity for a target of interest involves iterative rounds of affinity purification and amplification via a process termed *in vitro*-Systematic Evolution of Ligands by EXponential enrichment (SELEX) [14,15] (Figure 1; top panel). While *in vitro*-SELEX results in aptamers with affinities and specificities for their cognate targets comparable to those seen with antibodies, this process does not guarantee the identification of aptamers capable of recognizing their target in the context of the cell membrane and of internalizing into the target cell. We have recently described a novel cell-based selection strategy that we refer to as cell-internalization SELEX for isolating aptamers that internalize upon binding to their cognate receptor [16,17,18] (Figure 1; bottom panel). Cell-internalization SELEX has several advantages over other selection approaches for targeted therapeutic applications: (1) it favors the isolation of RNAs that bind to receptors in their native state; and (2) it enriches for RNAs capable of entering the target cell. To date, this approach has yielded aptamers capable of internalizing into HER2-positive mammary carcinoma cells [16], vascular smooth muscle cells [17] and TrkB-expressing cells [18]. Importantly, we demonstrated that when conjugated to therapeutic siRNAs, the cell-internalizing aptamers were capable of delivering their cargo to the cytoplasm of the target cells resulting in a robust RNAi response [16].

Although, the cell-internalization SELEX protocol has been useful at identifying several aptamers for siRNA-delivery [16,17], this approach does not necessarily select for aptamers that effectively escape endosomes and are released into the cytoplasm of target cells. Modifications to this approach (e.g., inclusion of fractionation steps to separate endosome-bound from cytoplasmic aptamer sequences) that allow for the selective amplification of cytoplasmic-specific aptamers may enable the effective isolation of cytoplasmic-targeting RNA sequences. The current cell-targeted aptamers for siRNA delivery must escape from early or late endosomes prior to entering the lysosome where the presence of nuclease and low pH conditions are likely to result in degradation of the aptamer-siRNA complexes [19]. Currently, neither the uptake kinetics of the cell-targeted aptamers nor the efficiency of release from endosomes is known.

Arguably, the future of this technology rests on the development of robust assays for the characterization, validation and optimization of cell-targeting aptamers as delivery agents. Here we describe several qualitative assays for assessing cell-specific internalization (microscopy-, microplate- and flow cytometry-based assays) and sub-cellular localization (RNA-RIP assay) of the RNAs. We also describe a novel quantitative assay (“QUSIM”) for measuring the efficiency of aptamer internalization. These methodologies promise to facilitate the development and characterization of cell-internalizing aptamers for delivery of therapeutics (e.g., siRNAs) into their target cells.

## 2. Results

### 2.1. Aptamers to Cell-Surface Receptors Analyzed with Binding/Internalization Assays

We assessed the advantages and disadvantages of five different assays—Microscopy Assay, Plate Reader Assay, Flow Cytometry Assay, Quantitative and Ultra-sensitive Internalization Method (*“QUSIM”*) and RNA-RIP Assay—using four previously described aptamers against cell-surface receptors [16,18,20,21] (Table 1). The first aptamer, A9g, is a 2'-fluoro-modified, truncated derivative of aptamer A9, which was isolated using the *in vitro-*SELEX protocol [22]. A9g binds prostate specific membrane antigen (PSMA) expressed on prostate cancer cells with high affinity and specificity. We have previously described the ability of A9g to target prostate specific membrane antigen (PSMA) expressing cells using RT-qPCR [20]. The second aptamer, E1, was the first aptamer isolated using the cell-internalization SELEX protocol (Figure 1; bottom panel) [16]. E1 was shown to internalize into cells which express the rat isoform of the HER2 receptor. This aptamer was tethered to Bcl2 siRNAs and evaluated as a potential therapeutic approach for sensitizing HER2-positive mammary carcinoma cells to chemotherapy [16]. The third aptamer, C4-3, is a 2'-fluoro-modified RNA aptamer to TrkB, which was also identified using the cell-internalization-SELEX protocol [18]. C4-3 binds the extracellular domain of TrkB with high affinity (*K_D_*∼2 nM) and exhibits potent TrkB partial agonistic activity and neuroprotective effects in cultured cortical neurons. Like A9g, the fourth aptamer, hHER2-apt, was isolated via the *in vitro-*SELEX protocol using a recombinant human HER2-GST fusion protein. Importantly, the authors confirmed binding specificity of hHER2-apt to HER2 expressed on human cancer cells [21].

**Table 1:** List of aptamers used in this study.

Aptamer ID	SELEX Method	Target	*K*_D_*(*nM*)*	Function	Reference
A9g	*In vitro*-SELEX	Human PSMA	5.0	Inhibition of PSMA (NAALADase) Enzymatic activity	[20]
E1	Cell-internalization SELEX	Rat HER2	60.8	N/A	[16]
C4-3	Cell-internalization SELEX	Mouse TrkB	~2.0	TrkB partial agonist	[18]
hHER2-apt	*In vitro*-SELEX	Human HER2	3.5	N/A	[21]

N/A: not addressed.

### 2.2. Assessment of Aptamer Internalization into Cells by Fluorescence Microscopy Assay

Methodologies for assessing cell-specific internalization of RNA aptamers are crucial to the development of powerful reagents capable of delivering therapeutics such as siRNAs or small molecule drugs to a target cell. Several approaches have been described by us [5,16,18,20,23,24] and others [7,8,9,22] to assess the internalization potential of these RNAs. These include quantitative approaches such as RT-qPCR and qualitative approaches such as fluorescence microscopy [5,16,17,18,20]. While RT-qPCR can help determine the efficacy of internalization of cell-specific aptamers, this approach is dependent on the efficiency of the wash step (to remove all surface bound RNA) and cannot distinguish between surface-bound RNAs and RNAs that have internalized into the target cells. Thus, RT-qPCR is often complemented with fluorescence microscopy to obtain more detailed information about the exact subcellular localization of the RNAs [8,11,16]. Fluorescence microscopy makes use of labeled-RNA aptamers, which are often chemically synthesized to include fluorescent dyes usually at their termini.

**Figure 2:**
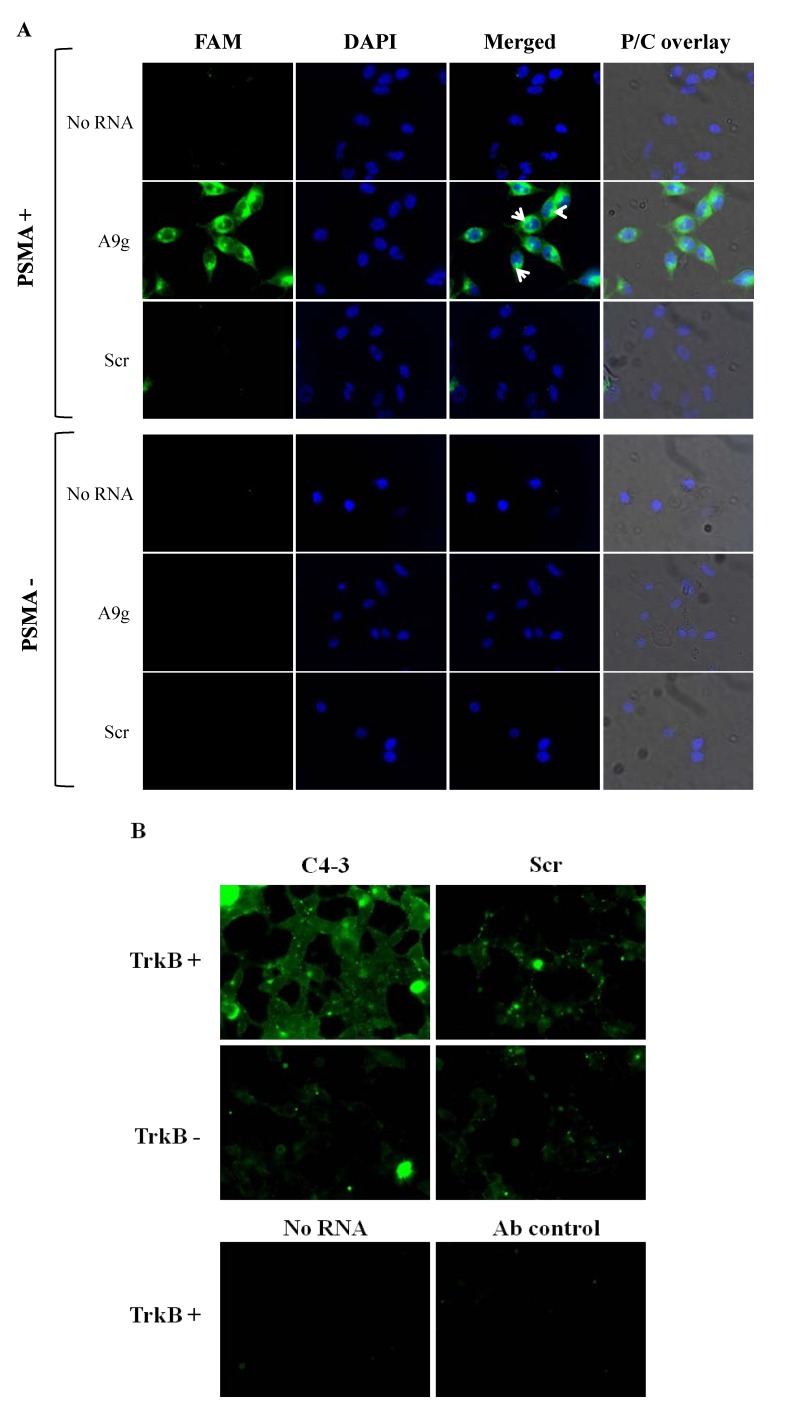
Fluorescence microscopy. (**A**) Direct fluorescence method. FAM labeled anti-PSMA RNA aptamer (A9g) was incubated with either PC3(PSMA+) or PC3(PSMA-) cells. A high salt wash step was performed to remove unbound or surface bound RNA. Internalized RNA was visualized using fluorescence microscopy. A scrambled, non-internalizing aptamer (Scr) was used as a negative control in these experiments. Florescence images were overlaid with DAPI and P/C (phase contrast) channels. Arrows indicate perinuclear localization of internalized A9g aptamer. (**B**) Antibody amplification method. FAM-labeled anti-TrkB RNA aptamer (C4-3) was incubated with either TrkB expressing or non-expressing HEK293 cells at 37 °C. FAM-labeled control aptamer (Scr) was used as a control for specificity. Unbound and surface bound RNA was removed as described above. Internalized RNA signal was amplified by incubation with an anti-FITC antibody and Alexa488 secondary antibody. Vehicle treated cells (No RNA) or cells subjected to incubation with antibodies alone (Ab control) were used as controls.

We evaluated the sub-cellular localization of FAM-labeled aptamer A9g using fluorescence microscopy (Figure 2A). FAM-labeled A9g was incubated with either PC3 cells lacking PSMA [PC3(PSMA-)] or PC3 cells expressing PSMA [PC3(PSMA+)] at 37 °C for 30 min (Figure 2A). A high salt wash step was used to remove unbound or surface bound RNA. As expected, fluorescence signal was observed only in PC3(PSMA+) cells. The signal was mostly localized in the perinuclear region, as shown by the arrow heads in the merged (FAM and DAPI) micrographs (Figure 2A). No signal was seen in PC3 cells lacking PSMA expression. Furthermore, a control, scrambled FAM-RNA (Scr) did not bind/internalize to PC3(PSMA+) cells, suggesting that the fluorescence signal is specific to A9g. Samples treated with buffer only (No RNA) were used to control for background fluorescence. We verified that the fluorescence signal seen in PC3(PSMA+) cells was mostly due to internalized RNA by performing the incubation of RNA on cells at 4 °C, which inhibits active transport (Supplementary Figure 1). As seen in Supplementary Figure 1, when the high salt wash step is applied to PC3(PSMA+) cells following incubation with FAM-A9g at 4 °C, the majority of the fluorescence signal is abrogated resulting only in a residual, low-intensity membrane signal (Supplementary Figure 1; right panel). This signal corresponds to surface bound FAM-A9g confirming that at 4 °C no active transport is occurring. Together, these data suggest that the fluorescence signal observed at 37 °C, in PC3(PSMA+) cells incubated with FAM-A9g, is due to internalized RNA.

Next, we modified the microscopy protocol to attempt to amplify the fluorescent signal. We refer to this protocol as the antibody amplification microscopy method. Antibody amplification microscopy method was employed to assess the sub-cellular localization of a different cell-specific aptamer (C4-3) previously shown to selectively internalize into TrkB-expressing cells [18]. Interestingly, when FAM-C4-3 was incubated with TrkB-expressing HEK293 cells, the fluorescence signal corresponding to internalized aptamer was difficult to discern (Supplementary Figure 2). A likely reason is that the direct method employing an aptamer labeled with a single FAM molecule is not sensitive enough when compared with typical immunofluorence labeling methods, in which several secondary antibodies, each labeled with 5-10 fluorophores, can bind a primary antibody, which binds the target protein. As a way to enhance the specific FAM-RNA signal, we employed antibodies to amplify the signal (Figure 2B). In this case, an anti-FAM primary antibody followed by an Alexa Fluor 488-labeled secondary antibody was employed to amplify the weak aptamer signal (Figure 2B). As shown, when the antibody amplification microscopy method is used, FAM-C4-3 signal can be discriminated from the background fluorescence signal. Binding specificity was confirmed by incubating TrkB-expressing cells with a scrambled RNA sequence (Scr) (Figure 2B; left panels). To control for cell auto-fluorescence or non-specific binding from the Alexa488-labeled antibody, TrkB-expressing cells were incubated with no RNA or secondary antibody only (Ab control) (Figure 2B, lower panels). In summary, we suggest that for certain aptamer sequences, an antibody amplification step may be necessary to determine cell-specific binding/internalization.

### 2.3. Assessment of Aptamer Internalization into Cells by Microplate Reader Assay

**Figure 3:**
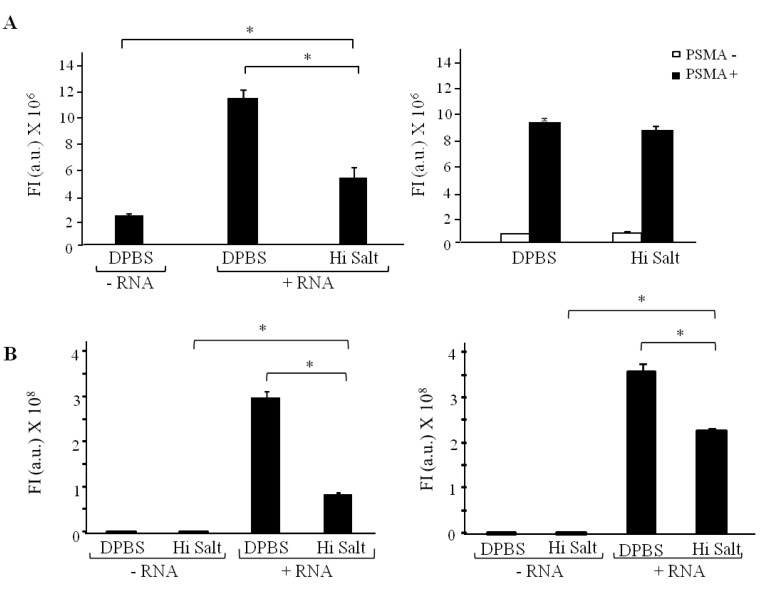
Plate-reader assay to assess aptamer binding and internalization. For the binding experiments, cells were fixed to inhibit active transport before incubation with the RNA aptamers. Live cells were used for the internalization experiments. (**A**) Binding (*left*) and internalization (*right*) of A9g into PSMA-expressing prostate cancer cells. (**B**) Binding (*left*) and internalization (*right*) of E1 aptamer into rat HER2-expressing mammary carcinoma cells. Cells only (no RNA) controls were carried out for each condition. Fluorescence was measured using an Analyst HT plate reader. (*, *p* < 0.001).

While microscopy approaches enable the sub-cellular visualization of fluorescently labeled RNAs they are often time consuming, requiring several incubation steps (as in the case of the antibody amplification method) and not amenable to high-throughput analysis. Microplates offer a convenient platform for high-throughput assay development, allowing for testing of a wide range of aptamers on several different cell-types, in an automated fashion. Here we describe a novel microplate-based fluorescence assay for determining cell-internalization of aptamers in a fast and reproducible manner. We evaluated the efficiency of this approach using two distinct aptamer sequences (A9g and E1) previously shown to undergo cell-specific internalization (Figure 3). Initially, we determined the binding (Figure 3A; left panel) and cell-internalization (Figure 3A; right panel) properties of the A9g aptamer. Binding studies were performed by incubating FAM-labeled A9g with fixed cells to block active uptake. A high salt wash step (0.5 M NaCl) was used to remove unbound and surface-bound RNA. As expected, we saw an increase in fluorescence in PSMA-expressing cells incubated with A9g compared to vehicle-treated cells (-RNA). Importantly, the fluorescence signal was greatly reduced when cells were subjected to the high salt wash step (Figure 3A; left panel). Cell-internalization studies were performed by incubating A9g on live cells at 37 °C. PSMA-lacking cells were used to control for specificity (white bars). As expected, A9g internalized preferentially in PSMA-expressing cells (black bars vs. white bars) (Figure 3A; right panel). Importantly, the fluorescence signal was predominantly due to internalized RNA given that the salt wash step minimally affected the fluorescence signal intensity. Together, these results suggest that the A9g aptamer internalizes efficiently into the target cells.

Next, we evaluated binding and internalization of aptamer E1 [16] to HER2-expressing cells (Figure 3B). As before, binding was determined by incubating FAM-labeled E1 with fixed N202.1A breast mammary epithelial cells expressing high levels of rat HER2 on the cell surface (Figure 3B; left panel). A high salt wash step was used to remove unbound or surface bound RNA. Interestingly, after performing the high salt wash step, we observed a significant residual amount of surface bound RNA. This result was different from that obtained with the A9g aptamer on PSMA-expressing cells and suggests that certain aptamer sequences may have a greater affinity for their target. It also highlights the need to optimize the wash step for each individual aptamer sequence. Although the high salt wash step failed to completely remove all surface bound E1 aptamer, when we assessed E1 aptamer internalization on live cells, the fluorescence intensity signal was considerably higher under these conditions compared to that of fixed cells. These results suggest that a significant (*p* < 0.001) amount of E1 aptamer does indeed internalize into the target cells. In addition, these results support our previous observations, that the E1 aptamer can be used to deliver functional siRNAs to ratHER2 expressing cells [16].

### 2.4. Assessment of Aptamer Binding/Internalization into Cells by Flow Cytometry

**Figure 4:**
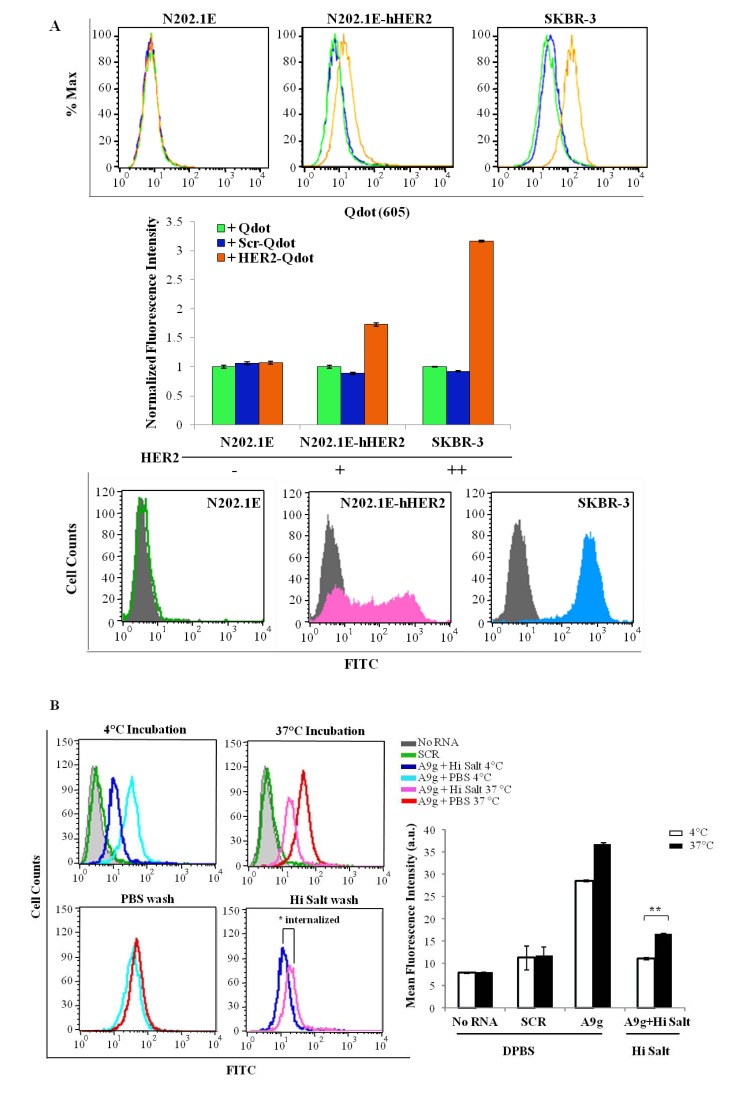
Evaluation of aptamer binding and internalization by flow cytometry. (**A**) Cell-specific binding of a human HER2 aptamer-Qdot conjugate. Cell lines expressing HER2 receptor (N202.1E-hHER2 and SKBR3) and HER2 non-expressing cell line (N202.1E) were incubated for 45min at 37 °C with human HER2 aptamer conjugated to Qdots (605nm). Cell-specific aptamer binding was evaluated by flow cytometry (upper panel). Quantification of specific fluorescence signal is shown in the middle panel bar graph. Cell surface human HER2 receptor expression in N202.1E, N202.1E(hHER2) and SKBR-3 cells (grey for isotype control, colored histograms for anti-HER2 Ab) (lower panel). (**B**) Measurement of A9g cell-internalization. PSMA-positive cells were incubated with FAM-A9g aptamer for 30 min at either 4 °C (left top panel) or 37 °C (right top panel). Cells were then washed with either DPBS or a High Salt (DPBS plus 0.5M NaCl) wash for 5min at 4 °C. The high salt wash step removes any unbound or surface bound aptamer. Bound and/or internalized aptamers were subsequently visualized using flow cytometry. *, internalized aptamer fraction (middle right panel). Fluorescence intensity quantified in the bar graph (**, *p* < 0.005).

We have previously used flow cytometry to characterize binding of fluorescently-labeled aptamers to their target cells [4,5,16]. Unlike the plate-reader method, which provides information on the amount of RNA bound to or internalized into the overall target cell population, flow cytometry can be used to determine binding/internalization on a cell-to-cell basis over a heterogeneous cell population. Here, we determined the ability of a previously reported aptamer directed against human HER2 [21] to bind to mammary carcinoma cell lines expressing high levels of human HER2 protein (Figure 4A). In this study, the human HER2 aptamer was conjugated to quantum dots (Qdots) via a 1:1 aptamer:biotin-streptavidin conjugation strategy. Quantum dots were selected for this approach given their greater fluorescence intensity over conventional fluorophores, which often require conjugation of multiple fluorophores to one aptamer leading to potential disruption of the aptamer structure. Specifically, a biotin-labeled human HER2 aptamer was coupled with streptavidin conjugated Qdots and incubated with either mouse mammary carcinoma cells (N202.1E) expressing hHER2 or with a HER2-positive human mammary carcinoma cell line (SKBR3). N202.1E cells (lacking HER2 expression) were used as controls for specificity. As predicted, a significant shift in fluorescence intensity, corresponding to specific human HER2 aptamer (hHER2-apt) binding to its protein target is observed (orange line) for both the N202.1E-hHER2 and SKBR-3 cells (Figure 4A; top panels). The normalized fluorescence intensity was plotted as a bar graph (Figure 4A; middle panel). No shift in fluorescence intensity was detected when the hHER2-apt was incubated with N202.1E cells lacking hHER2. Additionally, a scrambled RNA sequence (Scr, blue line) did not result in an increase in fluorescence intensity in any of the cell types tested, confirming the specificity of the hHER2-apt sequence for its target. Furthermore, the degree of fluorescence intensity observed in the N202.1E-hHER2 and SKBR-3 cells correlates with the amount of target protein expression on the cell surface (Figure 4A; bottom panels).

In addition to assessing binding of aptamers to the surface of cells, flow cytometry can be employed to assess internalization of RNA aptamers into target cells [4,16]. Next, we characterized the amount of FAM-labeled A9g internalized into PSMA-expressing prostate cancer cells. The reason for choosing a FAM-labeled RNA over the biotin-streptavidin Qdot conjugate is that the large size of the Qdot conjugate could interfere with cell uptake. FAM-labeled A9g was incubated with PC3(PSMA+) cells at 37 °C as described above to enable active cellular uptake. Incubation of cells at 4 °C was also performed to determine surface bound RNA and confirm the efficacy of the wash step. Unbound or surface bound RNA was removed by performing a stringent, high salt wash step at low pH (0.5M NaCl + 0.2N acetic acid). As anticipated, the stringent wash step efficiently removed surface bound RNA (compare stringent salt wash to DPBS wash) at both 4 °C (light and dark blue lines) and 37 °C (magenta and red lines) (Figure 4B; upper panel). The specific internalized fraction of A9g is given by the right shift in fluorescence signal (Figure 4B; lower right histogram) and was found to be 20% of the total mean fluorescence signal following the stringent wash step (Figure 4B; bar graph). Together, these results highlight the usefulness of this approach for assessing binding/internalization of aptamers to cells and for determining relative levels of target on the cell-surface.

### 2.5. Assessment of Aptamer Internalization into Cells by Quantitative & Ultra-Sensitive Internalization Method (“QUSIM”)

Near infrared (NIR) fluorophores provide superior sensitivity, low background auto-fluorescence and excellent performance for *in vivo* imaging. Additionally, the stability of NIR-labeling (under varying pH, temperature and light conditions) allows for real time tracking of molecular events. These properties of NIR fluorescence make it ideal for performing sub-cellular distribution and kinetic studies of various ligands.

**Figure 5:**
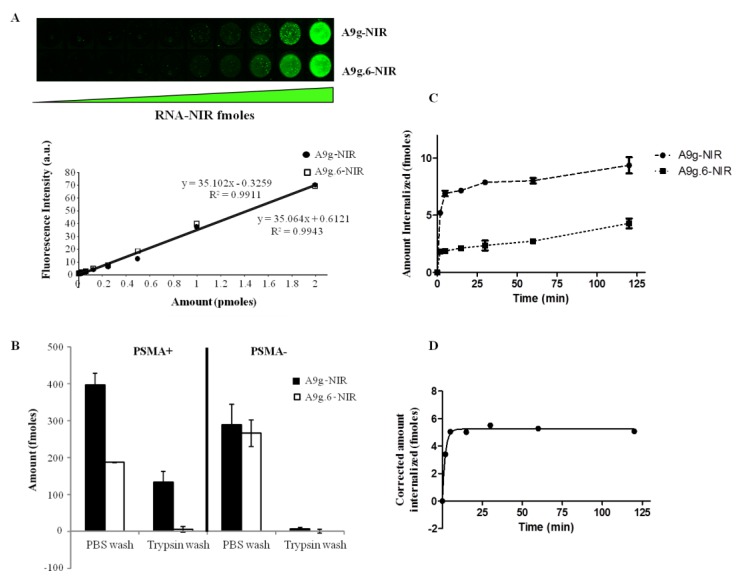
Quantitative and ultrasensitive internalization method (*“QUSIM”*). (**A**) 96-well microplate NIR Odyssey imager scan of serial dilutions for binding aptamer-NIR conjugate (A9g-NIR) and mutant, non-binding sequence conjugate (A9g.6-NIR) (*upper panel*) and standard curves with linear regression for RNA aptamer quantification (*lower panel*). (**B**) Quantification of the amount of aptamer-NIR internalized into PC3(PSMA+) cells *vs.* PC3(PSMA–) cells. (**C**) Time-dependent cell uptake of binding aptamer (A9g-NIR) vs. non-binding (mutant) aptamer (A9g.6-NIR). (**D**) Kinetics of specific A9g internalization using one-phase association curve fit (R^2^ = 0.9924, *k* = 0.542 min^−1^, half-time = 1.278 min).

Here we describe a novel, rapid and sensitive assay for assessing aptamer internalization, based on direct NIR-fluorescence detection of labeled-RNA aptamers using the Odyssey infrared imaging system from Li-Cor. To assess the usefulness of this approach for measuring cell-internalized RNA, we labeled the A9g aptamer with the NIR dye, IR800CW (Supplementary Figure 3A and B) and incubated the labeled RNA with either PC3(PSMA+) or PC3(PSMA-) cells (Figure 5). A mutant non-binding aptamer A9g.6 [20] was used as a control for specificity in these assays. First, we confirmed that coupling of the IR800CW dye to A9g does not affect aptamer function. This was done by evaluating the ability of NIR-A9g to inhibit PSMA enzymatic activity (NAALADase assay) (Supplementary Figure 3C). This assay provides a measure of the inhibitory activity of the A9g and thus, an indirect measure of A9g binding to PSMA [20]. Importantly, coupling of IR800CW to A9g did not affect aptamer function (Supplementary Figure 3C).

To quantitate the amount of A9g internalized in cells, we set up two-fold serial dilutions of labeled aptamers (A9g and A9g.6) and generated standard curves as shown in Figure 5A. Next, we incubated the A9g-NIR with either PC3(PSMA+) or PC3(PSMA–) cells in suspension for 15 min at 37 °C. Surface bound RNA was successfully removed by performing a trypsin wash step for 30min at 4 °C (Figure 5B). Based on this assay, we determined that approximately 13% (133 fmoles) of the input A9g aptamer internalized into PC3(PSMA+) cells after 15 min. In contrast, less than 0.5% of A9g.6 internalized into PC3(PSMA+) cells under the same conditions. Furthermore, internalization of A9g-NIR was specific for PC3(PSMA+) cells as less than 1% of the RNA internalized in cells lacking PSMA surface expression. Interestingly, when a PBS wash step was performed to assess surface bound plus internalized RNA, the background fluorescence signal was high and it was impossible to distinguish between RNA internalized into the target *vs.* the non-target cells. This result highlights the importance of optimizing the wash step for any given aptamer sequence when performing cell-based binding/internalization studies and suggests that the DPBS is not optimal for removing nonspecific binding. It also suggests that coupling of certain dyes (e.g. NIR) may render RNA more “sticky” resulting in higher non-specific binding.

In addition to performing quantitative measurements of internalized NIR-labeled aptamers, the *“QUSIM”* method can be used to measure kinetics of uptake of aptamers into the target cells. We determined the rate of A9g-NIR uptake into PC3(PSMA+) cells (Figure 5C and D). NIR-labeled A9g and A9g.6 aptamers were incubated with PC3(PSMA+) cells at 37 °C, over a 120 min period. At the end of the incubation period, cells were placed in ice to halt active transport. Cells were subjected to a trypsin wash for 30 min at 4 °C to remove unbound and surface bound RNA. A9g-NIR was taken up by cells as early as minutes. The amount of A9g internalized was calculated using a standard curve based on known amounts of A9g-NIR (Supplementary Figure 4) and was normalized to that of A9g.6 (considered to be non-specific uptake) (Figure 5D). We determined the rate constant of A9g uptake into PC3(PSMA+) to be 0.54 min^−1^. Peak internalization occurred at 5 min after which, the fluorescence internalized signal plateaued (Figure 5D). The reason for the plateau effect could be due to loss of the NIR label once the RNA is internalized or to slow membrane recycling of the PSMA receptor back to the cell surface. Indeed, it is possible that binding of A9g-NIR may lead to subsequent receptor degradation via an endocytic pathway. Together, these results suggest that *“QUSIM”* offers a rapid/quantitative platform for accurate quantification and assessment of kinetics of aptamer internalization.

### 2.6. Assessment of Aptamer Internalization into Cells by RNA-RIP (Ribosome Inactivating Protein) Assay

**Figure 6:**
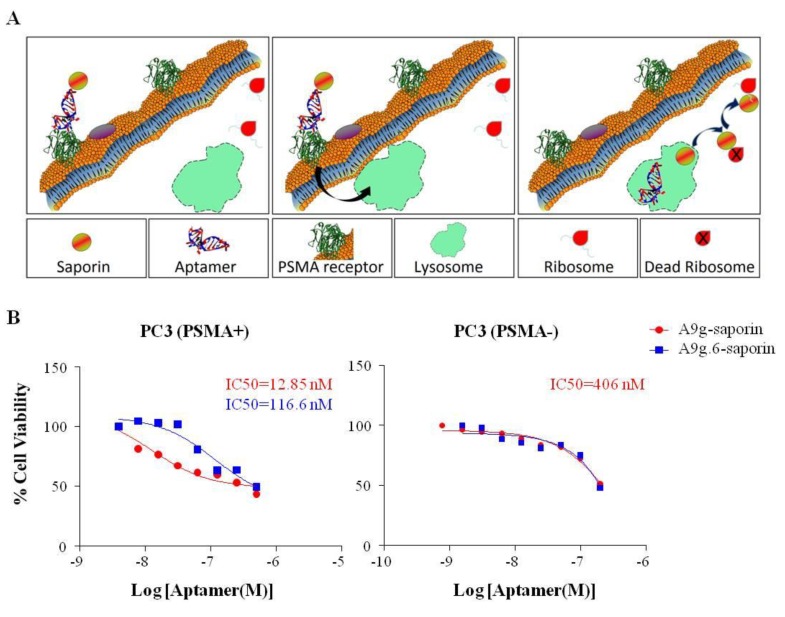
RNA-RIP assay. (A) Schematic of A9g-saporin conjugate internalization and RIP effect leading to cell death. (B) Dose dependent response of A9g-saporin and control, A9g.6-saporin, conjugates in PC3(PSMA+) (*left* ) and PC3(PSMA–) cells (*right*).

While the assays described above provide a reliable way of assessing cell-internalization of aptamers, they all rely on the efficiency of the wash step to distinguish between surface-bound RNA and RNA that has effectively internalized into the target cells. Functional assays, which link internalization to a functional readout (e.g., cell viability) would therefore, constitute a valuable and powerful alternative for fast and reliable screening of internalizing aptamers. Here, we describe a functional assay based on the cytotoxic properties of the saporin toxin. Saporin, a toxin derived from the plant *Saponaria officinalis*, irreversibly inhibits protein synthesis of eukaryotic cells when delivered into the cytoplasm of cells [25] (Figure 6A). Its ribosome inactivating protein (RIP) effect relies on its translocation from the extracellular environment to the cytoplasm of the cell [26]. Because saporin on its own is unable to penetrate cellular membranes, it is often conjugated to ligands, which mediate intracellular delivery (Figure 6A) [27,28,29,30,31,32,33]. We chose to make use of this property of saporin to evaluate internalization and cytoplasmic delivery of the A9g aptamer. Biotin-labeled A9g was conjugated to streptavidin-modified saporin (streptavidin-ZAP). First, we verified that conjugation of streptavidin-ZAP did not affect the inhibitory effect of the aptamer by performing the NAALADAse assay as described above (Supplementary Figure 5A). Next we evaluated the effect of A9g-saporin on PC3(PSMA+) (Figure 6B; left panel) and PC3(PSMA-) (Figure 6B; right panel) cells. Cells were treated with varying amounts of aptamer-saporin conjugate for 72 h at 37 °C after which, a viability assay (MTS assay) was performed to determine potential cytotoxicity of the conjugate. A non-targeting conjugate (A9g.6-saporin) was used as a control for specificity in this assay. As expected, cytotoxicity was more pronounced with the A9g-saporin conjugate (IC_50_ = 12.9 nM) than with the non-targeting conjugate (A9g.6-saporin; IC_50_ = 116.6 nM) (Figure 6B; left panel). In addition, little-to-no effect by either conjugate was seen in cells lacking PSMA expression (Figure 6B; right panel). Moreover, as a positive control for saporin cytotoxicity, we investigated the cytotoxic effects of a saporin-based conjugate (FGF-saporin) directed against the FGF (basic fibroblast growth factor)-receptor on PC3(PSM+) and PC3(PSMA-) prostate cancer cell lines (Supplementary figure 5B). The results suggest that PC3(PSMA-) cells are typically more sensitive to saporin toxicity further confirming the greater toxicity effect seen in PC3(PSMA+) cells compared to PC3(PSMA-) cells with A9g-saporin (Figure 6B; left panel). Together, these results confirm that A9g is being internalized preferentially into the target cells. Importantly, they also confirm that A9g is efficiently accessing the cytoplasm of target cells possibly through a mechanism of endosomal escape, resulting in inhibition of protein synthesis and ultimate cell-death. These findings are of importance and suggest that cell-internalizing aptamers, like A9g, can be used to effectively deliver therapeutics, such as siRNAs, directly to the RNA interference (RNAi) machinery in the cytoplasm.

## 3. Experimental

### 3.1. Cell Culture and Transfection

All cell lines were maintained in NuAire water-jacketed CO_2_ incubators at 37 °C with 5% CO_2._ N202.1A and N202.1E cells [34,35] were generously provided by G. Forni (University of Torino, Italy). N202.1A is a mammary carcinoma clonal cell line derived from a mammary tumor from the HER2/neu transgenic mouse of FVB background. N202.1A cells express high levels of surface HER2/neu, while the N202.1 E clonal cell line has no detectable surface expression of the HER2/neu oncoprotein. N202.1E-h HER2 cells were generated by stably transfecting N202.1E cell line with ErbB2-pHIV7-neo lentiviral construct using Polybrene, kindly provided by Al Klingelhutz (University of Iowa, USA). N202.1A (HER2^+^) and N202.1E (HER2^−^) cells were cultured in Dulbecco's Modified Eagle Medium (DMEM) supplemented with 20% fetal bovine serum (FBS, HyClone, Logan, UT, USA). N202.1E-h HER2 cells were maintained in medium supplemented with 200 μg/mL G-418. PSMA-non expressing prostate cancer cell line, PC3(PSMA-), and PSMA-expressing prostate cancer cell line, PC3(PSMA+), were maintained according to the supplier’s recommendations (ATCC #CRL-1435) in DMEM/F12 media with 10% FBS. PC-3(PSMA+) were derived from PSMA expressing PC-3 cells (generously provided by Eli Gilboa, University of Miami, USA) by clonally expanding a clone with high PSMA expression and further sorting using PSMA-PE labeled anti-PSMA antibody from MBL. HEK293 (ATCC cat# CRL-1573) and HEK293 cells expressing TrkB were maintained in Dulbecco's modified Eagle's medium supplemented with 10% fetal bovine serum. HEK293 cells were stably transfected with TrkB plasmid DNA by the lipofectamine method (Invitrogen, Grand Island, NY, USA). To achieve high-expressing TrkB cells, the cells were subjected to an anti-TrkB antibody recognizing the extra cellular domain (Chemicon cat# AB9872), followed by a secondary antibody conjugated to Alexa Fluor 488 (goat anti-rabbit AF 488, Invitrogen cat # A11008), as previously described [18]. SK-BR-3 breast adenocarcinoma cells (ATCC cat# HTB-30) were cultured in ATCC-formulated McCoy’s 5a Medium Modified (Cat# 30-2021) supplemented with 10% FBS. All cell lines were screened for contamination by mycoplasma, which is known to degrade 2′-fluoro pyrimidine modified RNA aptamers [36].

### 3.2. Receptor Cell Surface Expression

*PSMA expression.* PC3(PSMA+) and PC3(PSMA-) cells were grown in 60 mm cell culture dishes. On the day of the experiment cells were trypsinized (0.05% trypsin –EDTA), washed with DPBS and 2 × 10^5^ cells/mL were incubated with 1 mL Block Buffer (4% FBS in DPBS) for 30 min at 25 °C. Next, cells were incubated for 30 min at 25 °C with a PE-conjugated monoclonal antibody against human PSMA (Cat# K0142-5) (1:100 dilution in Block) or Block containing a control antibody-PE. Then, cells were washed twice with 500 μL Block, fixed in 500 μL FIX (1% formaldehyde in DPBS) and analyzed by flow cytometry. PC3(PSMA-) cells served as a negative control cell line in this experiment.

*HER2 expression.* N202.1E, N202.1A, N202.1E-h HER2, and SK-BR-3 cells were cultured as described above. Cells were washed one time with DPBS, pelleted and resuspended in 100 μL of 1:200 dilution (in Block) of primary antibody against either human HER2-Ab-2 (NeoMarkers, Grand Island, NY, USA; cat # MS-229-P1), rat HER2 Ab-4 (Calbiochem, Fremont, CA, USA; cat# OP16) or 1:500 dilution (in Block) of isotype-specific control antibody. After 30 min incubation at 25 °C, cells were washed twice with 500 µL Block and incubated with 1:500 secondary antibody (goat anti-mouse Alexa Fluor488 IgG; cat# A11001) for 20 min at 25 °C in the dark. Cells were washed as detailed above and analyzed by flow cytometry.

### 3.3. RNA Aptamers

Unless noted otherwise, all RNA aptamer sequences were chemically synthesized with 2′ fluoropyrimidine (italics)–modified nucleotides obtained from TriLink Biotechnologies (San Diego, CA). Depending on the type of assay/application, aptamers were chemically synthesized with either 3'- or 5'-FAM, 5'-NH2 or 5'-biotin groups.

*A9g aptamer*

5'-GGGA*CC*GAAAAAGA*CCU*GA*CUUCU*A*U*A*CU*AAG*UCU*ACG*UUCCC*-3'

*A9g.6.aptamer*

5'-GGGACCGAAAAAGACCUGGCUUCUAUACUAAGUCUACGUUCCC-3'

*hHER2 aptamer*

5'-AG*CC*G*C* GAGGGGAGGGA*U*AGGG*U*AGGG*C*G*C*GG*CU*-3'

*E1 aptamer*

5'‐GGGAGGA*C*GA*U*G*C*GG*U*CC*U*G*UC*G*UCU*G*UUC*G*UCCCC*AGA*C*GA*CUC*G*CCC*GA‐3'

*Scr aptamer*

5'‐GGGAGGA*C*GA*U*G*C*GGGA*CU*AG*C*GA*UCU*G*UU*A*C*G*C*A*C*AGA*C*GAC*UC*G*CCC*GA‐3'

*C4-3 aptamer*

5'-GGGAGGA*C*GA*U*G*C*GG*UC*G*U*A*UU*A*UCC*G*CU*G*C*A*C*G*C*CAGA*C*GA*CUC*G*CCC*GA-3'

*Scr2 aptamer*

5'-GGGAGGA*C*GA*U*G*C*GG*UUU*GGGG*UUUUCCC*G*U*G*CCCC*AGA*C*GA*CUC*G*CCC*GA-3'

### 3.4. Fluorescence Microscopy

*Direct method*. For internalization experiments, PC3(PSMA+) and PC3(PSMA-) cells were grown on 35 mm glass-bottom MatTek culture dishes in DMEM/F12 media supplemented with 10% FBS. Cells were seeded at a density of 2 × 10^5^ cells/mL in a volume of 500 μL one day prior to the experiment. On the day of the experiment, cells were washed with pre-warmed DPBS buffer and incubated for 30 min at 37 °C with 100 μL of 500 nM FAM-labeled aptamers (FAM on the 5'end) A9g (5'-GGGA*CC*GAAAAAGA*CCU*GA*CUUCU*A*U*A*CU*AAG*UCU*ACG*UUCCC* -3') or control aptamer with a scrambled sequence: (5'‐GGGAGGA*C*GA*U*G*C*GGGA*CU*AG*C*GA*UCU*G*UU*A*C*G*C*A*C*AGA*C* GAC*UC*G*CCC*GA‐3'). One hundred μL of DPBS alone was added to cells as no RNA control in these experiments. Next, cells were washed twice with ice-cold DPBS adjusted to 0.5 M NaCl (high salt wash) and 0.2 N acetic acid to remove unbound and surface bound RNAs. Subsequently, nuclei were stained with DAPI (1 g/mL) at room temperature for 15 min followed by washing three times with DPBS. For surface binding experiments (validation of the high salt wash experiments) cells were initially pre-conditioned by washing them with ice-cold DPBS. Subsequently, cold aptamer solutions or DPBS were incubated on cells at 4 °C for 10min. Nuclei were further stained as previously described and cells mounted for microscopy visualization.

*Antibody amplification method*. The antibody amplification protocol was carried out essentially as described previously [18] with a few modifications. Briefly, HEK293 cells and TrkB-expressing HEK293 cells were grown on glass-bottom MatTek culture dishes in DMEM plus 10% FBS. Staining was performed as previously described [18]. Cells were washed with DPBS pre-warmed at 37 °C following a 1 h incubation at 37 °C with FAM-labeled (FAM was on 3'end) C4-3 or control aptamer (100 nM RNA in 100µL) in DPBS. A non-specific, non-binding aptamer was used as a control in these experiment (Control aptamer: 5'-GGGA GGA*C*GA*U*G*C*GG*UUU*GGGG*UUUUCCC*G*U*G*CCCC*AGA*C*GA*CUC* G*CCC*GA-3'.

Following incubation with the RNA, cells were washed twice with ice-cold DPBS and then for 5 min with 0.5 M NaCl and 0.2 N acetic acid at 4 °C. Cells were then fixed for 20 min at room temperature with 4% formaldehyde, 4% sucrose in DPBS. The cells were permeabilized with 0.1% Triton X-100 in DPBS for 5 min. A block was performed by incubating cells with 5% goat serum plus 0.1% Triton X-100 in DPBS for 1 h at room temperature. The fluorescence signal was amplified by incubating with an anti-FITC antibody (rabbit anti-FITC, 1:1000 dilution; Invitrogen). The primary antibody incubation was followed by incubation with a fluorescent secondary antibody (goat anti-rabbit IgG, Alexa Fluor 488 conjugate, 1:1000 dilution; Invitrogen). Cells were mounted and coverslipped with a glycerol mounting medium.

All images were acquired with a 40X oil immersion objective, an Olympus IX71 inverted microscope, a cooled CCD camera and filters for FITC (excitation, 450–490 nm; emission, 515–565 nm). Data is representative of at least three captured images *per* condition. For the experiments with A9g aptamer, intracellular localization was further confirmed by overlaying the fluorescence images with the nuclear DAPI stain and subsequently, with the phase/contrast images using ImageJ 1.46 d software. The fluorescence images reported are representative of at least three captured images *per* dish/*per* condition. All microscopy experiments were repeated at least twice in different days.

### 3.5. Fluorescence Plate Reader

PC3(PSMA+), PC3(PSMA-) or N2021.A cells (expressing rat HER2) were grown to confluency in 150 mm culture dishes. Cells were then trypsinized and transferred to 1.5 mL Eppendorf tubes, in triplicate for each condition. Cells were centrifuged at 500 g for 3 min. Media was discarded and the cells were washed once with pre-warmed DPBS. For the binding experiments, cells were then fixed with 4% formaldehyde, 4% sucrose for 20 min at room temperature to inhibit internalization of aptamers. For the internalization experiments the fixation step was eliminated. Cells were further incubated with 100 µL of FAM-labeled chemically synthesized 2'fluoro RNAs (200 nM A9g, or 1 µM E1) for 1 h at 37 °C. After the RNA incubation, cells were subjected to either a DPBS wash to remove unbound aptamer, or a high salt wash (0.5 M NaCl) to remove unbound aptamers and aptamers bound to the cell membrane. For the high salt wash condition, cells were washed once briefly with both ice-cold DPBS and 0.5M NaCl, followed by a 5 min incubation with ice-cold 0.5M NaCl. Cells were further rinsed once with 1 mL DPBS and lysed using 400 µL of 0.1 N NaOH. Next, 100 µL of each lysate was loaded in triplicates onto a 96 well plate. Fluorescence was measured at 485/530 nm on an Analyst HT plate reader from LJL. At least two individual experiments *per* aptamer were performed using this assay.

### 3.6. Flow Cytometry

Binding of Qdot-aptamer conjugates. To prepare the Qdot-aptamer conjugates, 2.5 μL of 2 μM streptavidin-modified Quantum dots 605 (Invitrogen, cat# Q10001MP) were incubated with chemically synthesized, either 3'-biotin human HER2 aptamer (5'-AG*CC*G*C*GAGGGGAGGGA*U*AG GG*U*AGGG*C*G*C*GG*CU*-3') or 5'-biotin control aptamer (5'-GGG*CC*GAAAAAGA*CCU*GA*CUUCU* A*U*A*CU*AAG*UCU*A*C*G*UCCC*-3') (497.5 μL of 50 nM) for 30 min at 25 °C under gentle stirring. The final Qdot-aptamer conjugates were washed by centrifugal spin filtration, resuspended in DPBS, and then characterized using gel electrophoresis. Cells (N202.1E, N202.1E-hHER2 and SB-BR-3) were grown to confluency in 60 mm dishes in their respective media. In the day of the experiment, cells were harvested with 0.25% trypsin-EDTA (N202.1E and SK-BR-3) or 0.05% trypsin-EDTA (N202.1E-h HER2), washed once with media containing 10% FBS and further blocked in 10% FBS in DPBS for 30 min at room temperature. Next, cells were incubated with Qdot-hHER2 conjugate, Qdot-control aptamer conjugate or free Qdots for 45 min at 37 °C. After washing twice with 10% FBS in DPBS, cells were resuspended in DPBS and analyzed by flow cytometry using a Becton Dickinson LSR II equipped with a 633 nm laser for Qdot excitation.

*Internalization of A9g-FAM*. PC3(PSMA+) positive cells were cultured as described previously. Cells were trypsinized, washed twice with DPBS and 2 × 10^5^ cells/mL aliquots prepared for internalization studies. Next, cells were incubated with 100 μL of 1 μM of either target aptamer (FAM-A9g), control aptamer (FAM-Scr) or DPBS for 30 min at either 4 °C (binding) or 37 °C (binding and internalization). Subsequently, cells were subjected to a DPBS wash or a high salt wash (0.5 M NaCl + 0.2 N acetic acid) to remove unbound and surface bound RNAs. The high salt wash was performed in two steps, as detailed above. This procedure ensures that majority of the surface bound sequences are removed, so that the detected signal is an accurate measure of specifically internalized RNAs. Finally, cells were suspended in DPBS and analyzed by flow cytometry.

### 3.7. Quantification

Quantification of aptamer internalization was determined using IRDye800-labeled A9g (NIR-A9g). Specifically, 5x10^4^ cells of either PC3(PSMA+) of PC3(PSMA-) cells were incubated with 100 μL of 10 nM of either target A9g-NIR or control (non-binding) A9g.6-NIR for 15 min at 37 °C. Cells were then washed with 500 μL of DPBS at room temperature or 100 μL of 0.25% trypsin-EDTA at 4°C for 30 min (to remove surface bound sequences). Cells that underwent trypsin wash were further washed two more times with 500 μL DPBS and finally, all samples were resuspended in 100 μL DPBS and transferred onto wells of a 96 well plate. Calibration curves for both, A9g-NIR and A9g.6-NIR, were constructed by measuring a series of twelve 1:2 dilutions starting from 10 nM for each aptamer. After sample loading, the plate was transferred to an Odyssey IR-Scanner (Li-COR, St. Lincoln, NE, USA) and scanned at 800 nm excitation using the supplied predefined settings for 96-well plate. For the time course studies, PC3 (PSMA+) cells were trypsinized with 0.05% trypsin-EDTA, washed with media containing 10% FBS and then resuspended in pre-warmed, serum free, DMEM/F12 media. Twenty thousand cells were incubated at 37 °C with A9g-NIR or A9g.6-NIR for the following time points: 120, 60, 30, 15, 5 min and no RNA sample serving as 0 time point. Internalization was halted by quickly transferring the samples to ice. Next, cells were incubated with 100 μL of 0.25% trypsin for 30 min at 4 °C to remove the surface bound RNAs, followed by a DPBS wash and then resuspended in 100 μL DPBS. Samples were loaded in 96 wells plate and scanned as described above. Aptamer uptake was time-dependent, showing an apparent exponential increase to a plateau value. Internalized aptamer data was fitted to a one-phase exponential association curve as described previously [37] using GraphPad Prism Software (San Diego, CA, USA).

### 3.8. IRDye800CW Conjugated Aptamers(A9g-NIR and A9g.6-NIR)

A9g and A9g.6 aptamers, chemically synthesized with a 5'-NH2 group, were incubated with IRDye800 CW NHS ester (Li-Cor, cat# 929-70021) at a ratio of 1:40 (RNA:dye) for 2 h at 25 °C in 1 M potassium phosphate buffer (pH 9) under gentle stirring. Unreacted dye was removed by five successive spin filtrations. Aptamer labeling with IR dye was confirmed by ESI spectrometry and UV-Vis spectrophotometry. Functionality (binding) of the aptamer-NIR conjugates was tested by the PSMA NAALADase activity assay as described below.

### 3.9. PSMA NAALADase Activity Assay

The PSMA NAALADase activity assay was performed as previously described [38] with minor modifications. Briefly, the final reaction volume was modified to 200 μL. Double-distilled H_2_O (ddH_2_O) was used for all solutions. The RNA aptamers were refolded in binding buffer (20 mM HEPES, 150 mM NaCl, 2 mM CaCl_2_) at 1 μM concentration. Refolding was accomplished by heating at 65 °C for 10 min, followed by slow cooling to 37ºC for 10 min. Aptamers were concentrated through an Amicon 10,000 MW-cutoff spin filter (Cat# UFC801024). The remaining procedures were performed on ice. Ten microliters of refolded RNA in binding buffer was combined with 50 μL of 200 mM Tris buffer, pH 7.5, and 20 μL 10 mM CoCl_2_. Recombinant PSMA was prepared by diluting 2 μg recombinant human PSMA (4234-ZN-010) from R&D Systems (Minneapolis, MN, USA) in 500 μL of 50 mM pH 7.5 Tris buffer. Ten microliters of the recombinant PSMA solution (40 ng PSMA) was added to the reaction mixture, and the reaction was incubated for 15 min at 37 °C to promote RNA-PSMA interaction. Ten microliters of a working solution containing 30 nM NAAG having a specific activity of 2.5 μCi/μL of [glutamate-3,4-3H]-NAAG from Perkin Elmer (Waltham, MA, USA; cat# NET1082250UC) was added to the reaction mixture. The reaction was allowed to proceed for 15 min, mixing once by gentle pipetting. The reaction was subsequently stopped by adding an equal volume (200 μL) of ice-cold 0.1M Na_2_HPO_4_ buffer. AG 1-X8 formate resin (200-400 mesh) from Bio-Rad (Hercules, CA, USA; cat# 140-1454) was used in columns to quantitate the [3H]-glutamate reaction product. Before use, the columns were equilibrated with 2 mL of ddH_2_O. Half of the final reaction volume (200 μL) was added to a column. The columns were eluted with 4 mL of 1 M formic acid and added to 10 mL of Bio-Safe II scintillation fluid (Research Products International Corp., Mt. Prospect, IL, USA). Activity was counted using a Beckman-Coulter liquid scintillation counter, and was normalized to the amount of activity obtained in the reaction with no RNA added.

### 3.10. RNA-RIP (Ribosome Inactivating Protein) Assay

Chemically synthesized, 5'-biotin RNA aptamers were reacted with a chemical conjugate of streptavidin and saporin toxin (Streptavidin-ZAP, Advanced Targeting Systems, San Diego, CA, USA; cat#IT-27) at room temperature for 30 min at a 1:1 molar ratio. The conjugates were characterized using gel electrophoresis. PSMA binding of aptamer-saporin conjugates was verified by PSMA NAALADase activity assay. PC3(PSMA-) and PC3(PSMA+) were seeded in 96 well plates (5 × 10^3^ cells/well), grown overnight and then treated with different concentrations (1:2 dilution series) of A9g-saporin, A9g.6-saporin or FGF (basic fibroblast growth factor)-saporin (FGF-sap, Advanced Targeting Systems; cat #IT-38) for 72 h at 37 °C. The viability of treated cells was evaluated with Promega Cell Titer 96 AQueous One Solution Cell Proliferation Assay (Promega, Madison, WI, USA). Briefly, 20 μL of Cell Titer 96 AQueous One Solution was added to each well, including four wells containing only medium for background deduction. Cells were then incubated at 37 °C for 90 min. Absorbance at 490nm in each well was then determined using a Molecular Devices (Sunnyvale, CA, USA) ThermoMax microplate reader.

## 4. Conclusions and Discussion

In this study, we describe both qualitative and quantitative assays for characterizing the cell-type specific internalization ability of RNA aptamers for cell-targeted therapeutic applications. These assays include qualitative assays such as: (1) antibody amplification microscopy, (2) microplate-based fluorescence assay and (3) quantum-dot-based flow cytometry as well as a quantitative assay that we refer to as quantitative and ultrasensitive internalization method (*“QUSIM”*). In addition, we describe a novel way to monitor for cytoplasmic delivery using a ribosome inactivating protein-based assay (RNA-RIP assay). Since translocation of the siRNA to the cytoplasm is a crucial aspect of aptamer-targeted siRNA delivery, assays for monitoring translocation to the cytoplasm, like the RNA-RIP assay, are of importance. The next challenge will be to develop similar approaches capable of providing not only a qualitative, but also, a quantitative measure of the portion of the cell-associated siRNA that reaches the cytoplasm and is processed by the RNAi machinery.

Despite initial excitement and promise in clinical application of RNAi-based drugs, recent setbacks have tempered the excitement for these therapeutics and have highlighted the need for extensive efforts from both Industry and Academia to overcome these key hurdles. Effective cell-specific targeting and cytoplasmic delivery of siRNAs are two of the main limitations for the successful translation of this technology to humans. In recent years, RNA aptamers have emerged as potentially clinically useful and effective cell-targeting ligands for RNAi-based drugs [39,40,41,42,43]. Importantly, these studies have also described a role for aptamers in facilitating delivery of siRNAs to the cytoplasm of target cells [4,5,7,9,10,11,12,13]. While, the precise mechanism for this is not well understood, it is generally accepted that efforts towards optimizing uptake into cells or escape from endosomes (two bottlenecks in intracellular delivery) would greatly enable the broad clinical application of this technology. Indeed, optimization of aptamer-mediated delivery of RNAi-based drugs will likely not only enhance efficacy and safety, but might also reduce the drug concentration and cost needed for effective therapy.

Methods for monitoring cellular uptake of cell-specific aptamers are the first step in optimizing reagents for targeted RNAi delivery. Many aptamer detection approaches involve adding multiple tags (fluorophores, biotin, *etc*.) to the body of the aptamer. One downside of these approaches is that they risk disrupting the aptamer structure. The alternative, which we prefer, involves end-labeling the aptamer with a single tag. However, in its simplest form (direct labeling: 1 tag/1 aptamer), this approach suffers from low sensitivity. To address this shortcoming, we have: (1) developed an antibody amplification method for the microscopy protocol, (2) coupled aptamers to quantum dots which are much brighter than conventional fluorophores (flow cytometry assay), (3) coupled aptamers to NIR dyes, which provide enhanced sensitivity compared to conventional fluorophores (“*QUSIM”* assay) and (4) coupled aptamers to a cellular toxin (saporin), which provides a sensitive cellular readout (RNA-RIP assay). As indicated above, the RNA-RIP assay not only provides a measure of cellular uptake but allows investigators to distinguish between those aptamers that internalize, but remain sequestered in endosomes and are eventually degraded by the lysosomal pathway, and those aptamers that escape the endosomes and are delivered to the cytoplasm of target cells where they engage into the RNAi pathway. This information may lead to further optimizations including attaching small molecules or combining with nanocarriers to enhance cellular uptake or endosomal escape.

In summary, the assays described herein provide powerful tools for assessing/confirming the internalization potential and sub-cellular localization of RNA aptamers within a target cell. Moreover, we show that the choice of assay to be performed depends largely on the nature of the aptamer sequence or that of its target receptor and suggest variations to protocols (see microscopy assay methodologies) for optimal sensitivity. Importantly, these efforts promise to expedite the development of RNA aptamer-based approaches for delivering siRNAs to the cytoplasm of target cells and possibly facilitate a more rapid translation of these therapeutics to humans.
